# JNK Pathway Mediates Low Oxygen Level Induced Epithelial–Mesenchymal Transition and Stemness Maintenance in Colorectal Cancer Cells

**DOI:** 10.3390/cancers12010224

**Published:** 2020-01-16

**Authors:** Shing Yau Tam, Vincent W.C. Wu, Helen K.W. Law

**Affiliations:** Department of Health Technology and Informatics, Faculty of Health and Social Sciences, The Hong Kong Polytechnic University, Hong Kong, China; shing-yau.tam@connect.polyu.hk

**Keywords:** Akt, colorectal cancer, epithelial–mesenchymal transition, hypoxia, JNK, oxygen level, stemness maintenance

## Abstract

(1) Background: Epithelial–mesenchymal transition (EMT) and cancer cell stemness maintenance (SM) are important factors for cancer metastasis. Although hypoxia has been considered as a possible factor for EMT induction and promotion of SM, studies in this area, apart from hypoxia-inducible factor (HIF) pathways and severe hypoxia, are scant. This study aimed to evaluate the effects of different oxygen levels on EMT induction and SM and elucidate the signaling pathways involved in colorectal cancer cells. (2) Methods: Cell morphological analysis, migration assay, immunofluorescence staining of cytoskeleton and Western blotting were performed on human colorectal cancer cells HT-29, DLD-1, and SW-480 cultured at 1%, 10%, and normal (21%) O_2_ levels. The role played by c-Jun N-terminal kinase (JNK) was evaluated through the use of the specific JNK inhibitor SP600125. (3) Results: This study evaluated 1% and 10% O_2_ are possible conditions for EMT induction and SM. This study also demonstrated the partial relieve of EMT induction and SM by SP600125, showing the importance of the JNK pathway in these processes. Furthermore, this study proposed a novel pathway on the regulation of Akt by JNK-c-Jun. (4) Conclusions: This study suggests 10% O_2_ as another possible condition for EMT induction, and SM and JNK pathways play important roles in these processes through multiple factors. Inhibition of JNK could be explored as treatment for inhibiting metastasis in colorectal cancer cells.

## 1. Introduction

Metastasis is an important event in cancer progression and accounts for majority of cancer related deaths including colorectal cancer (CRC) [1]. Although much of the exact mechanism remains unknown, epithelial–mesenchymal transition (EMT) has been regarded as an important event for metastasis as it allows cancer cells to transform from the closely packed epithelial cell type to mesenchymal cell type with migratory and invasive properties [2].

The loss of adherent junctions between cells alters the cytoskeletal composition and cell polarity to form spindle-shaped cells. In cancer cells undergoing EMT, the actin cytoskeleton is reorganized from cortical thin bundles into thick contractile stress fibers at the ventral cell surface [3]. The monomers of actin polymerize to form filamentous-actin (F-actin) and start the formation of various migratory protrusions including podosomes, invadopodia, filopodia, and lamellipodia. This process, known as dynamic actin reorganization, is a prerequisite for the migration and invasion of cancer cells [4,5].

Hypoxia induces EMT mainly by hypoxia-inducible factor (HIF) activation [6]. HIF-1α could bind directly to twist-related protein 1 (TWIST1), matrix metalloproteinase-9 (MMP-9), and histone deacetylase 3 (HDAC3), which eventually promote transcription of Snail [7]. Meanwhile, HDAC3 regulates the formation of histone methyltransferase complexes to induce vimentin and N-cadherin. For CRC, ubiquitin-specific protease 47 (USP47) reduced E-cadherin expression by Snail regulation under hypoxic conditions [8], and the overexpression of TWIST1 and Snail is associated with poor prognosis [9].

Apart from EMT of the primary tumor, cancer stem cells (CSCs) have been widely accepted as precursors of metastases [10]. CSCs are pluripotent for multilineage differentiation, capable for self-renewal, and have long longevity [11]. They vary in number among different types of cancer cell lines and human xenografts, affecting tumor differentiation and aggressiveness [12]. CSC proliferation and maintenance of stemness properties are controlled by similar pathways as EMT-inducing pathways such as Wnt, transforming growth factor-β (TGF-β), and Notch [13]. The pathways exert their influences on stemness maintenance (SM) by regulating pluripotency markers including octamer-binding transcription factor 4 (Oct-4), sex determining region Y-box 2 (SOX2), and Nanog. In CRC cells, these pluripotency markers are regarded as poor prognosis indicators of CRC. In CRC stem cells, aberrant Wnt/β-catenin signaling is observed, and this is suggested to cause treatment resistance and disease relapse [13].

Although HIFs have been suggested to influence EMT and stemness though multiple pathways under hypoxic conditions, the influence of other hypoxia-related molecules such as c-Jun N-terminal kinase (JNK) has received little attention [6,13]. JNK is activated by various stresses and was initially regarded as an inducer of inflammation and apoptosis. However, JNK could also be activated by chronic hypoxia and influence autophagy [14]. JNK may influence EMT though TGF-β SMAD-independent pathway [6] and SM through Wnt/bone morphogenetic protein (BMP)/T cell factor (TCF)/Notch or interleukin 6 (IL-6)/Janus kinase (JAK)/signal transducer and activator of transcription 3 (Stat3) pathways [15].

Cancer cells constantly face different oxygen level during development from hypoxia to about 10% oxygen level during metastasis in the bloodstream. In our previous autophagy study, we have reported that 10% oxygen level could exert prominent effects on oxygen level driven regulators including HIFs and JNK [14]. Therefore, the impact of various oxygen levels on EMT and SM was further investigated in this study.

## 2. Results

### 2.1. Low Oxygen Levels Induced Morphological Signs of EMT, Which Was Reversed by JNK Inhibition

**Figure 1:**
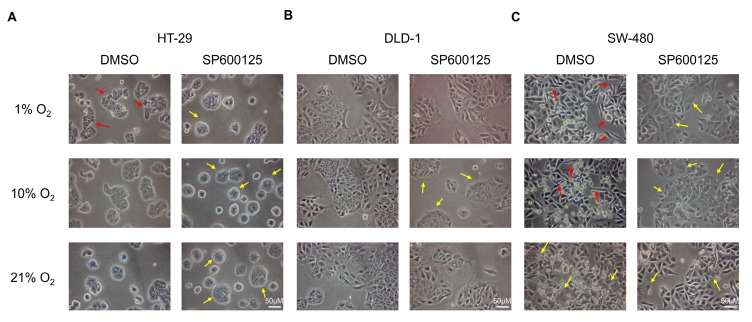
Cell morphological changes under different oxygen levels. HT-29 acquired more loosely packed colonies (red arrows) under 1% O_2_ conditions, while the presence of SP600125 (10 µM) changed the cell colony to be more rounded and more densely packed (yellow arrows) under all oxygen levels investigated (**A**) DLD-1 did not show prominent changes in cell morphology under different oxygen level incubation. Cell colonies became more closely packed in 10% O_2_ conditions (yellow arrows) in the presence of SP600125 (**B**) SW-480 showed more mesenchymal features at 1% and 10% O_2_ by having more spindle-shaped cells (red arrows). More epithelial cell colonies (yellow arrows) formed under different oxygen levels with SP600125 addition, especially in 1% and 10% O_2_ conditions (**C**). Sample images from 5 independent experiments performed (200× magnification).

From the photomicrographs taken after 48 h incubation, the three CRC cell lines (HT-29, DLD-1, and SW-480) showed different responses to different oxygen levels. For HT-29, which had more epithelial cell features among the three cell lines, cells became more loosely packed colonies under 1% O_2_ (Figure 1A), showing signs of EMT induction in the loss of cell–cell interactions [16]. Also, cell colonies incubated under 1% and 10% O_2_ became more irregularly shaped from the dominantly round shape in 21% O_2_. In the presence of JNK inhibitor, the cell colony shape in all three oxygen levels became rounder, similar to the control, when compared with those without the addition of JNK inhibitor.

For DLD-1, which had intermediate epithelial and mesenchymal cell features among the three studied cell lines, there was no prominent changes of morphological features under different oxygen levels (Figure 1B). Whereas, in the presence of JNK inhibitor, cell colonies of DLD-1 cultured in 10% O_2_ became more closely packed.

SW-480 normally had the most mesenchymal cell features among the three cell lines. In the 1% and 10% O_2_ conditions, they acquired larger cell colonies of spindle-shaped cells when compared with cells in 21% O_2_, showing signs of EMT induction (Figure 1C). In the presence of JNK inhibitor, the EMT induction in 1% and 10% O_2_ were partially reversed by having more epithelial cell colonies and less spindled-shaped mesenchymal-like cells.

### 2.2. Cell Migration Was Reduced by JNK Inhibition

**Figure 2:**
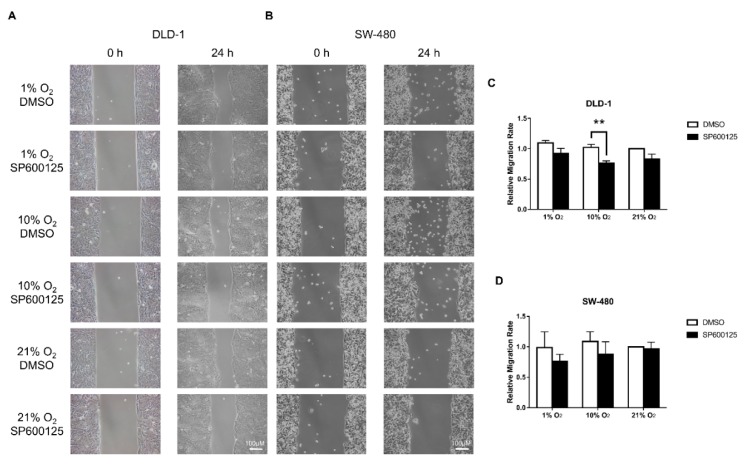
Wound healing assay under different oxygen levels. Wound healing assay showed that there were no significant differences of migration rate between different oxygen level incubation in DLD-1 (**A**,**C**) and SW-480 (**B**,**D**) cells. While the presence of JNK inhibitor SP600125 (10 µM) generally reduced the migration rate among different oxygen levels in both cell lines, with 10% O_2_ having significant reduction in DLD-1. The data (means ± SEM) were expressed as the relative migration rate against the rate under 21% O_2_ + DMSO (**C**,**D**). ** *p* < 0.01, DMSO versus SP600125. *N* = 3 (SW-480) and 4 (DLD-1).

Cell migration was investigated by wound healing assay. Preliminary experiments using HT-29 showed an extremely slow migration rate, probably due to their epithelial properties (Supplementary Figure S1); hence, they have been excluded in the analysis. Results for DLD-1 and SW-480 showed that there was no significant difference in migratory rate when cells were cultured in different oxygen levels (Figure 2). Whereas, the addition of JNK inhibitor generally mildly reduced the migratory rate among different oxygen levels. A significant reduction of migratory rate at 10% O_2_ in DLD-1 was found.

### 2.3. JNK Inhibition Reduced Lamellipodia and Filopodia Formation

**Figure 3:**
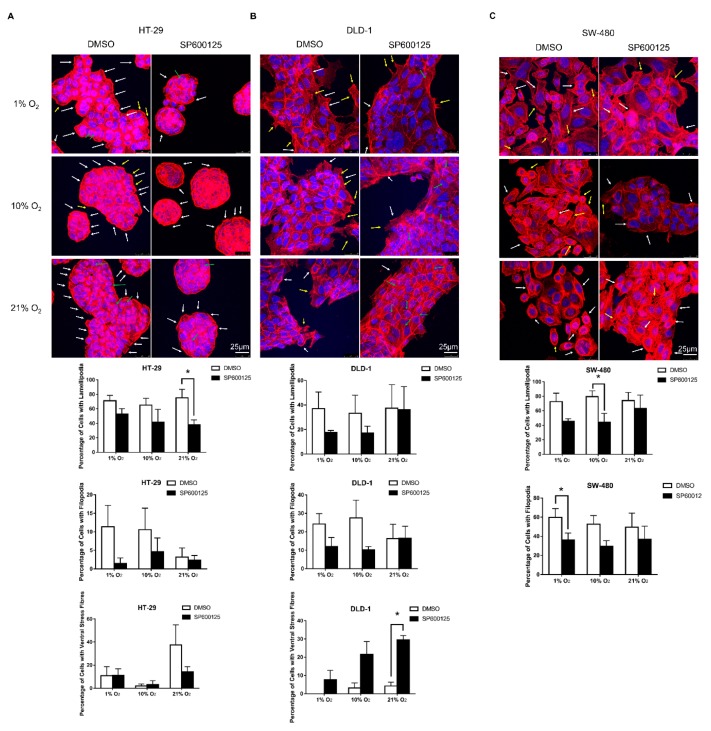
Immunofluorescence staining of F-actin under different oxygen levels. Immunofluorescence staining of F-actin (red) and DAPI (blue) showed an increase in filopodia formation (yellow arrows) and a decrease in ventral stress fiber formation (green arrows) in 1% and 10% O_2_ conditions, while lamellipodia formation (white arrows) was similar among different oxygen levels in HT-29 (**A**) DLD-1 also showed an increase in filopodia formation in 1% and 10% O_2_ with similar lamellipodia formation among different oxygen levels (**B**) SW-480 showed similar amounts of lamellipodia and filopodia formation among different oxygen levels, and ventral stress fibers were not observed in SW-480. (**C**) The presence of JNK inhibitor SP600125 slightly reduced filopodia and lamellipodia formation among the three cell lines, while ventral stress fiber formation was promoted in DLD-1. Sample images were taken from 3 independent experiments. The counting results were shown as means ± SEM in percentage among all evaluated cells. At least 200 cells were evaluated for each sample. * *p* < 0.05 DMSO versus SP600125. *N* = 3.

Immunofluorescence staining of F-actin by phalloidin was conducted to evaluate the actin cytoskeleton. For HT-29, majority of cells had lamellipodia formation (Figure 3A). There was a trend of increased filopodia formation at lower oxygen levels. Ventral stress fibers are described as thick and directed contractile fibers [3]. There was a decreasing percentage of cells with ventral stress fibers with lowering oxygen levels. JNK inhibition reduced the formation of lamellipodia, filopodia, and ventral stress fibers in different oxygen levels (Figure 3A).

For DLD-1, a smaller population of cells had lamellipodia formation when compared with HT-29, while a similar increase of filopodia formation in lower oxygen levels was observed (Figure 3B). JNK inhibition could slightly reduce lamellipodia and filopodia formation in 1% and 10% O_2_ but generally promoted ventral stress fiber formation in DLD-1.

In SW-480, similar percentages of cells with lamellipodia and filopodia formations were recorded among different oxygen levels (Figure 3C). The presence of JNK inhibitor reduced formations of lamellipodia and filopodia. There was no observable ventral stress fiber formation in SW-480.

### 2.4. EMT Induced by Low Oxygen Levels via JNK Pathway Was Confirmed by EMT Markers and Transcription Factors

To further investigate the underlying signaling pathways in leading to these morphological and cytoskeleton changes and the role of JNK in EMT and stemness pathways, the expression of related proteins including EMT markers, EMT transcription factors, JNK pathway markers, other EMT related pathway markers, and SM markers were evaluated by Western blotting.

**Figure 4:**
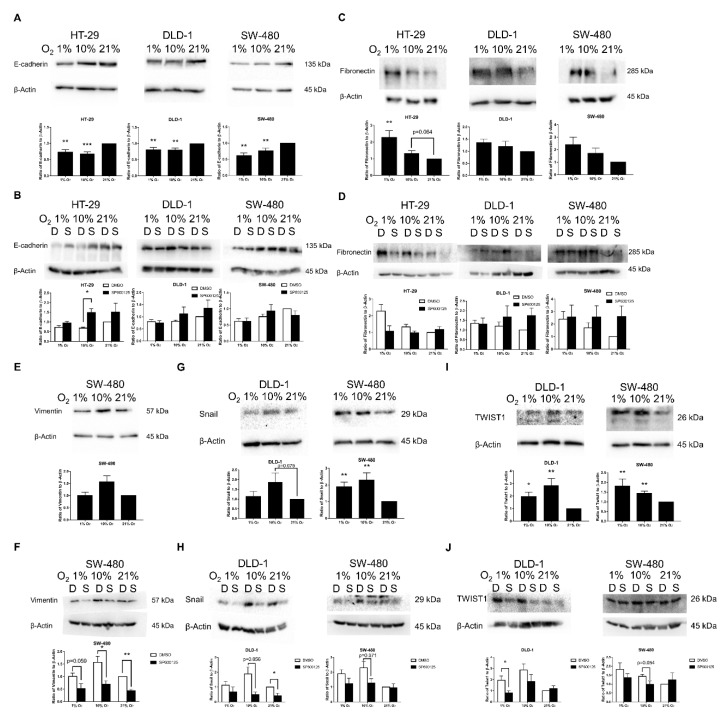
Epithelial–mesenchymal transition (EMT) marker and transcription factor expressions under different oxygen levels. EMT markers and transcription factors were evaluated by Western blotting in different oxygen levels on HT-29, DLD-1, and SW-480 cell lines. The key epithelial EMT marker E-cadherin was significantly down-regulated in all three studied cell lines in 1% and 10% O_2_ (**A**) JNK inhibitor SP600125 (10 μM) could generally up-regulate E-cadherin in different oxygen levels, especially for 10% O_2_ in HT-29 (*p* < 0.05) (**B**) The mesenchymal EMT marker fibronectin was generally up-regulated in lower oxygen levels with 1% O_2_ in HT-29 having significant up-regulation (**C**) HT-29 experienced down-regulation of fibronectin in the presence of SP600125, while DLD-1 and SW-480 had up-regulation of fibronectin in 10% and 21% O_2_ by SP600125 (**D**) Another mesenchymal EMT marker, vimentin, was only detectable in SW-480; it was mildly promoted in 10% O_2_ but not in 1% O_2_ (**E**) SP600125 could substantially reduce vimentin expression under different oxygen levels, especially for 10% and 21% O_2_ (**F**) EMT transcription factors Snail and TWIST1 expression levels in different oxygen levels were evaluated in DLD-1 and SW-480 (**G**–**J**) Substantial promotions of Snail in lower oxygen levels were found with SW-480 having significant increase in 1% and 10% O_2_ (**G**) Snail was generally suppressed by SP600125 with more prominent effects in DLD-1 (**H**) TWIST1 also had significant promotion in lower oxygen levels among SW-480 and DLD-1 (**I**) and it experienced a suppression effect by SP600125 in both 1% and 10% O_2_ (**J**) The data (means ± SEM) were expressed as the relative expression compared with 21% O_2_ group, *N* = 9 (HT-29 and DLD-1) or 10 (SW-480) (**A**,**C**,**E**,**G**,**I**) The data (means ± SEM) were expressed as the relative expression compared with 21% O_2_ + DMSO group. *N* = 9 (HT-29 and DLD-1) or 10 (SW-480) for DMSO group, *N* = 4 (HT-29) or 5 (DLD-1 and SW-480) for SP600125 group (**B**,**D**,**F**,**H**,**J**). * *p* < 0.05, ** *p* < 0.01, *** *p* < 0.001. D: DMSO (0.1%), S: SP600125 (10 μM).

The key EMT marker, E-cadherin, was significantly down-regulated in 1% and 10% O_2_ in all three studied cell lines (Figure 4A). JNK inhibitor SP600125 (10 μM) could generally promote E-cadherin in different oxygen levels, especially for 10% O_2_ in HT-29 (Figure 4B).

The mesenchymal marker fibronectin was generally promoted in 1% and 10% oxygen levels among the three cell lines with significant promotion in 1% O_2_ on HT-29 (Figure 4C). The impact of SP600125 on fibronectin had diverged responses among the three cell lines (Figure 4D). Down-regulation of fibronectin in HT-29 was found in lower oxygen levels, whereas DLD-1 and SW-480 experienced up-regulation trends in 10% and 21% O_2_.

Another mesenchymal marker, vimentin, was only detectable in SW-480. It was mildly promoted in 10% O_2_ but not in 1% O_2_ (Figure 4E). JNK inhibitor reduced vimentin expression under different oxygen levels (Figure 4F).

EMT transcription factors Snail and TWIST1 were also investigated in DLD-1 and SW-480 cells. Both Snail (Figure 4G) and TWIST1 (Figure 4I) were up-regulated in 1% and 10% O_2_ among both cell lines, with SW-480 having significant promotions in both Snail and TWIST1 at lower oxygen levels, while DLD-1 could attain significant increase in TWIST1. Both Snail (Figure 4H) and TWIST1 (Figure 4J) were generally inhibited by JNK inhibitor in DLD-1 and SW-480, especially for DLD-1 in lower oxygen levels.

### 2.5. Confirmation of JNK and Akt Pathway Activation in Low Oxygen Levels

**Figure 5:**
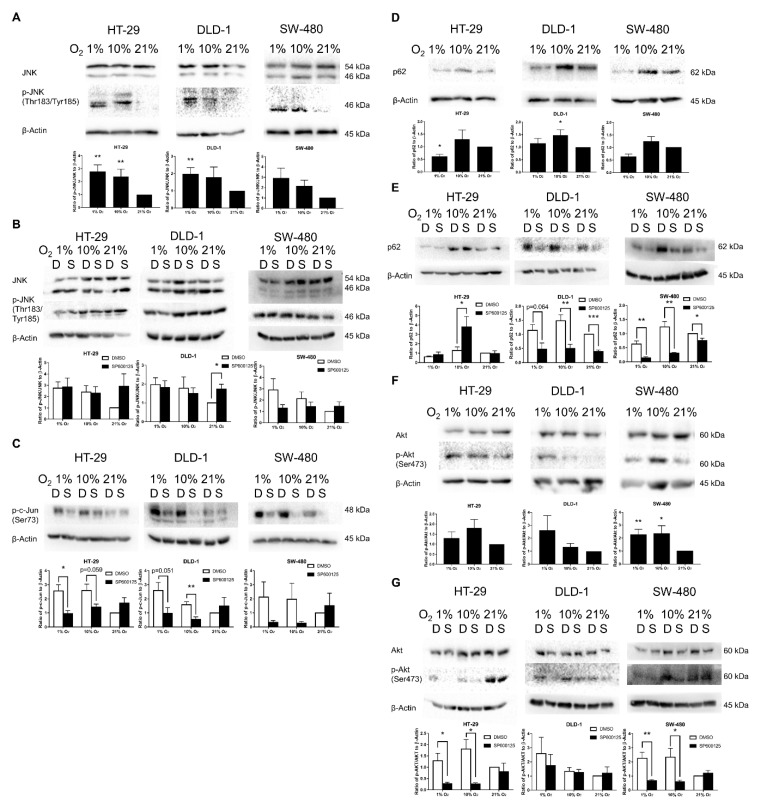
JNK and Akt pathway activation under different oxygen levels. The key markers of JNK pathways were evaluated by Western blotting. JNK was activated under lower oxygen levels in HT-29, DLD-1, and SW-480 with significant promotion in HT-29 and DLD-1 (**A**) There were generally mild reductions in JNK phosphorylation status by SP600125 (10 μM) in 1% and 10% O_2_ among the three cell lines, with greater effects on SW-480, while JNK phosphorylation was slightly promoted in 21% O_2_ (**B**) For the JNK direct downstream target c-Jun, its phosphorylation status p-c-Jun has been largely suppressed by SP600125 in lower oxygen levels, with significant reductions found in HT-29 and DLD-1 (**C**) The indirect downstream effector of JNK pathway p62 showed mild up-regulation in 10% O_2_ and down-regulation in 1% O_2_ (**D**) In the presence of SP600125, significant down-regulations were found in different oxygen levels among DLD-1 and SW-480 (**E**) However, for HT-29, significant up-regulation of p62 was evaluated in 10% O_2_. Akt, which is another important EMT promoter, also demonstrated an increase in activation under lower oxygen levels in the three cell lines, with SW-480 having significant activation (**F**) The 1% and 10% O_2_ activated Akt was abruptly inhibited by SP600125, particularly in HT-29 and SW-480 (**G**) The data (means ± SEM) were expressed as the relative expression compared with 21% O_2_ group, *N* = 9 (HT-29), 8–9 (DLD-1), or 10 (SW-480) (**A**,**D**,**F**). The data (means ± SEM) were expressed as the relative expression compared with 21% O_2_ + DMSO group. *N* = 4–9 (HT-29), 5–9 (DLD-1), or 5–10 (SW-480) for DMSO group; *N* = 4 (HT-29) or 5 (DLD-1 and SW-480) for SP600125 group (**B**,**C**,**E**,**G**). * *p* < 0.05, ** *p* < 0.01, *** *p* < 0.001. D: DMSO (0.1%), S: SP600125 (10 μM).

JNK and Akt pathways are possible regulators of EMT transcription factors. It was confirmed that JNK phosphorylation in CRC was detected in both 1% and 10% O_2_ conditions for the three studied cell lines, with significant activations in HT-29 (1% and 10% O_2_) and DLD-1 (10% O_2_) (Figure 5A). JNK inhibitor did not directly affect the phosphorylation status of JNK (Figure 5B). However, it suppressed the direct target of JNK c-Jun by inhibiting the phosphorylation status of p-c-Jun (Figure 5C) in different oxygen levels among all three studied cell lines.

For the indirect downstream effector of JNK pathway, p62 had diverse changes under lower oxygen levels with general promotion at 10% O_2_ and general down-regulation at 1% O_2_ (Figure 5D). JNK inhibitor suppressed p62 in DLD-1 and SW-480 and activated p62 in 10% O_2_ of HT-29 (Figure 5E). This evidence demonstrated the successful general JNK inhibition.

For Akt phosphorylation, all three cell lines showed general activation under lower oxygen levels with statistically significant up-regulation in SW-480 (Figure 5F). For the parallel EMT-inducing Akt pathway, JNK inhibition could abruptly inhibit low oxygen level activated Akt, with statistically significant suppression found among HT-29 and SW-480 (Figure 5G).

### 2.6. Promotion of SM under Low Oxygen Levels Was Partially Reversed by JNK Inhibition

**Figure 6:**
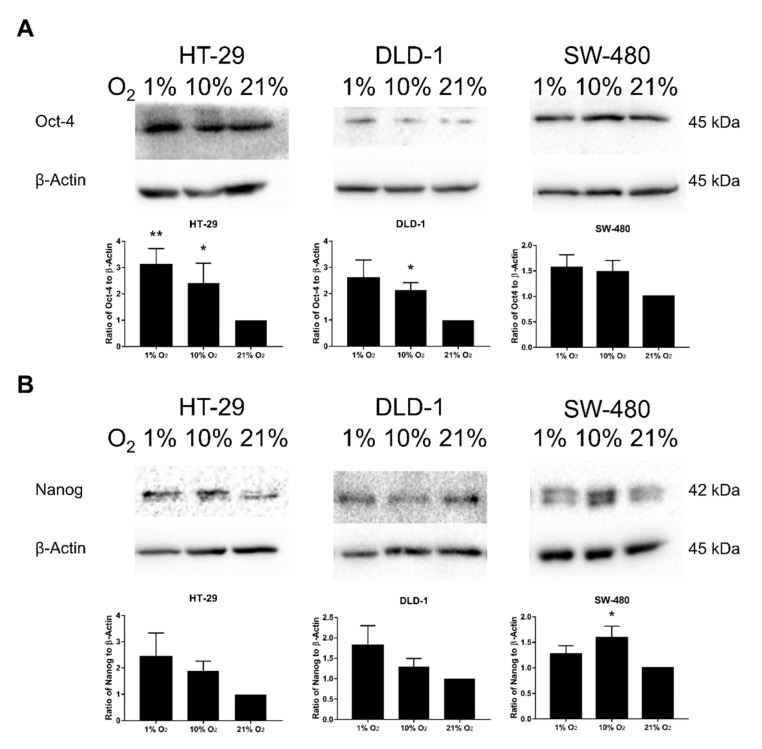

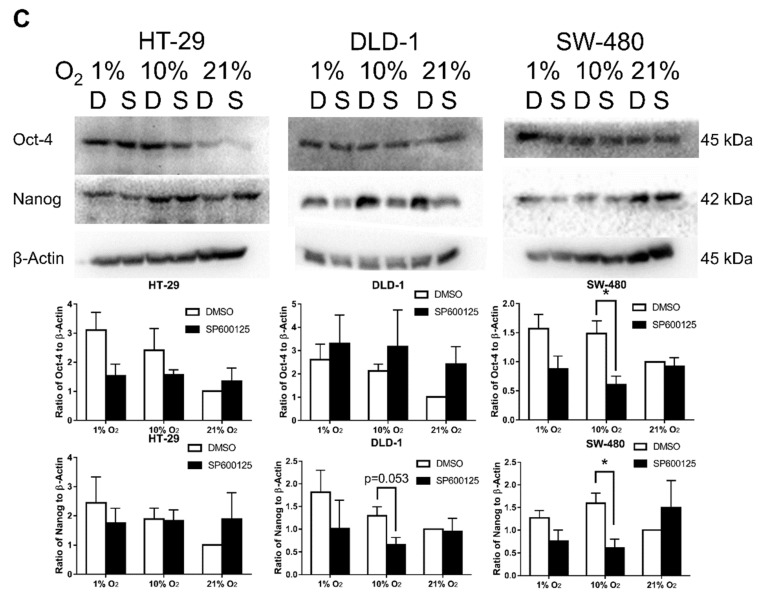
Oct-4 and Nanog expressions under different oxygen levels. The key stemness maintenance markers Oct-4 (**A**) and Nanog (**B**) changes in lower oxygen levels were evaluated by Western blotting. Results showed that both stemness maintenance markers were generally up-regulated under lower oxygen levels with significant up-regulation of Oct-4 in HT-29 (1% and 10% O_2_) and DLD-1 (10% O_2_) and Nanog in SW-480 (10% O_2_). For the impact of JNK inhibitor SP600125 (10 μM) on Oct-4 (**C**), diverse impacts with general down-regulation in lower oxygen levels among HT-29 and SW-480 and slight up-regulation in DLD-1 by SP600125 were found. While for Nanog, general inhibition in 1% and 10% O_2_ was discovered among all three studied cell lines with greater impact on DLD-1 and SW-480 (**C**). The data (means ± SEM) were expressed as the relative expression compared with 21% O_2_ group, *N* = 9 (HT-29 and DLD-1) or 10 (SW-480) (**A**,**B**) The data (means ± SEM) were expressed as the relative expression compared with DMSO 21% O_2_ group. *N* = 9 (HT-29 and DLD-1) or 10 (SW-480) for DMSO group; *N* = 4 (HT-29) or 5 (DLD-1 and SW-480) for SP600125 group (**C**) * *p* < 0.05, ** *p* < 0.01. D: DMSO (0.1%), S: SP600125 (10 μM).

Changes of SM markers of CSC, including Oct-4 and Nanog, in different oxygen levels were also studied. Results revealed general promotion of both markers in lower oxygen levels with greater promotion of Oct-4, especially for HT-29 and DLD-1 (Figure 6A,B). Significant up-regulations were found at Oct-4 in HT-29 (1% and 10% O_2_) and DLD-1 (10% O_2_) and Nanog in SW-480 (10% O_2_). For the study of the effect of JNK inhibition on Oct-4 and Nanog (Figure 6C), diverged results had been discovered in Oct-4 among the three cell lines. Oct-4 was generally suppressed by JNK inhibition among HT-29 and SW-480, while DLD-1 experienced slight up-regulation instead. In the meantime, Nanog was generally down-regulated by JNK inhibition among all three cell lines in 1% and 10% O_2_.

## 3. Discussion

EMT has been regarded as an important event for metastasis as it transforms cancer cells from the relatively stationary epithelial cell type to invasive mesenchymal cell type [2]. From the results of 48 h of incubation, lower oxygen levels (1% and 10% O_2_) induced morphological changes in CRC cells, and the cells became less organized as the cell–cell separation increased, especially for 1% O_2_. This demonstrated the loss of cell–cell contact, which is an initial step for EMT. In the presence of JNK inhibitor, dramatic changes of cell morphology were observed, and JNK inhibition could reverse the situation by inducing pro-epithelial cell morphology changes.

Cancer cells undergoing EMT may have increased invasiveness and migratory power. The wound healing assay evaluated the impact of lower oxygen levels and JNK inhibition on the migratory rate of CRC cells. Because of the poor migratory rate of HT-29, only DLD-1 and SW-480 were analyzed. We did not detect a significant difference of relative migratory rate in different oxygen levels, probably due to the time limitation of this simple wound healing assay, which could only provide relatively accurate data within 24 h as cell division may affect the assay reliability, and EMT is a relatively slow process. Moreover, the high confluence of cell culture required in a wound healing assay could reduce the effectiveness of EMT induction [17]. Nevertheless, the presence of JNK inhibitor reduced the migratory rate in different oxygen levels, further supporting the notion that JNK promotes CRC migration. Apart from the macroscopic changes in cell morphology and migration, we also demonstrated EMT induction based on the cytoskeleton changes due to dynamic actin reorganization. Actin polymerization could occur by increasing F-actin formation to promote the formation of various migratory structures including lamellipodia, filopodia, and stress fibers and the inhibition of migratory structure formation by JNK inhibitor.

Epithelial marker E-cadherin and mesenchymal markers fibronectin and vimentin are important EMT markers. The loss of E-cadherin was confirmed in all three CRC cell lines in 1% and 10% O_2_. Although hypoxia (1% O_2_) has been described in previous research as a factor of EMT induction, the significant loss of E-cadherin in 10% O_2_ found in this study is a novel finding. E-cadherin expression was enhanced in the presence of JNK inhibitor with greater enhancement in 10% O_2_ and in HT-29. This suggests the general relieve of EMT induction by JNK inhibition. Moreover, the ineffective relieve in 1% O_2_ suggests the activation of other EMT-inducing pathways, such as the HIF pathway, while the predominant EMT-inducing pathway in 10% O_2_ is JNK-mediated.

In line with the observation of EMT-related morphological changes, our results showed that fibronectin was generally up-regulated under lower oxygen levels, especially in HT-29 and SW-480. While, for another mesenchymal marker, vimentin, its expression level was observable only in SW-480. This suggests that SW-480 had the highest mesenchymal status among the three cell lines studied. Results found that vimentin was generally up-regulated in 10% O_2_ but not for 1% O_2_. Vimentin is usually promoted by HIF-1α-HDAC3 in hypoxia [6], but this promotion was not seen in 1% O_2_ samples in this study. It may be due to the short-lived nature of HIF-1α, while the promotion trend in 10% O_2_ may be due to another pathway that was activated at a low oxygen level. In the presence of JNK inhibitor, vimentin was significantly suppressed. The trends of the results in mesenchymal markers coincided with E-cadherin results, as HT-29 achieved better relieve of EMT by JNK inhibitor. Moreover, the results suggested JNK mediation of vimentin.

Various EMT transcription factors have been proposed among different cancer types, including Snail, Slug, TWIST1/2, and zinc finger E-box-binding homeobox 1/2 (ZEB1/2), as they may affect transcription of E-cadherin and other EMT markers [6]. Among the three cell lines studied, only Snail and TWIST1 could be detected in DLD-1 and SW-480 by Western blotting. This indicates the higher mesenchymal statuses of DLD-1 and SW-480 than that of HT-29. In HT-29, the low expression of Snail may be due to low expression of HDAC3 [18]. Snail binds to the promoter of CDH1 to repress E-cadherin transcription and therefore promote EMT [6]. The highest expression level of Snail in SW-480 among the three cell lines probably is due to the highest expression of HDAC3 [18,19], in which HDAC3 could promote Snail by HIF-1α-HDAC3-Snail pathway. From the results of this study, SW-480 had significant up-regulation of Snail in both 1% and 10% O_2_, while DLD-1 only had substantial activation in 10% O_2_ but not for 1% O_2_. The difference of activation in 1% O_2_ mainly is due to the difference of activation between SW-480 and DLD-1 by HIF-1α-HDAC3-Snail pathway. Although HIF-1α-HDAC3-Snail may account for the activation of Snail in 1% O_2_, HIF-mediated EMT induction could not account for Snail up-regulation in 10% O_2_ and there should be another pathway leading to the up-regulation of Snail [20].

TWIST1 belongs to the basic helix-loop-helix (bHLH) transcription family, which is involved in cancer metastasis and flanks the CDH1 gene to repress E-cadherin [6]. TWIST1 is found to be a direct target of HIF-1α, rather than as an indirect target like Snail [6]. Our results demonstrated significant up-regulation of TWIST1 in both the HDAC3-deficient DLD-1 and HDAC3-activated SW-480. Similar to Snail, TWIST1 was also significantly up-regulated in 10% O_2_ for both DLD-1 and SW-480, demonstrating activation by another EMT-inducing pathway other than HIF-1α under low oxygen level conditions, as HIF-1α does not activate in 10% O_2_ [20]. Both Snail and TWIST1 confirmed EMT induction in lower oxygen levels. Moreover, both Snail and TWIST1 were inhibited by JNK inhibition in both DLD-1 and SW-480.

In this study, we have evaluated JNK pathway related proteins including JNK, its indirect down-stream regulator p62, and its possible up-stream regulator Akt. The JNK pathway is a possible delayed pathway that is activated in 10% O_2_ in light of our previous study [14]. Also, there was research showing that JNK signaling may contribute to Snail and TWIST1 activation by DNA methyltransferase 1 (DNMT1) or TGF-β1-induced EMT pathway by promoting fibronectin and vimentin [21,22,23,24,25,26,27]. Our results demonstrated the general activation through hyperphosphorylation under low oxygen levels among all three cell lines, and this confirmed the activation of JNK pathway among CRCs.

p62 is an autophagy adaptor protein that may bind to various EMT regulators including TWIST1, mothers against decapentaplegic-4 (SMAD4), and vimentin [28,29,30]. Our results showed general down-regulation of p62 in 1% O_2_ while opposite results were found in 10% O_2_. Down-regulation of p62 in 1% O_2_ may be due to the promotion of autophagy under severe oxidative stress for cell survival [31], and this correlates with the lower expression of vimentin in SW-480 under 1% O_2_ versus 10% O_2_. While the up-regulation of p62 in 10% O_2_ corresponds to the activation of JNK-c-Jun-p62 and could contribute to the up-regulation of TWIST1 and vimentin in 10% O_2_.

Consistent with the past study on the effect of SP600125 on a variety of kinases and enzymes [32], SP600125 did not inhibit the phosphorylation of JNK but rather by inhibiting its downstream targets such as c-Jun. Results of p-c-Jun expression in the three cell lines demonstrated the effective inhibition by SP600125, especially at low oxygen levels. The results of p-c-Jun and EMT transcription factors suggest the possible JNK-c-Jun-Snail/TWIST1 pathway for EMT induction under low oxygen levels. Moreover, fibronectin and vimentin down-regulation by SP600125 found in HT-29 suggests possible activation of JNK-c-Jun-Fibronectin/vimentin pathway under low oxygen levels. While for the indirect JNK target p62, SP600125 could achieve significant inhibitions for DLD-1 and SW-480 in different oxygen levels. However, SP600125 could up-regulate p62 expression in HT-29 instead. The down-regulations of p62 and Snail/TWIST1 in DLD-1 and SW-480 suggest another possible JNK-mediated EMT-inducing pathway by JNK-c-Jun-p62-Snail/TWIST1 under low oxygen levels.

Akt, which is usually seen as an activator of EMT and an inhibitor of autophagy through phosphoinositide 3-kinase (PI3K)-Akt-Snail/Slug and PI3K-Akt-mechanistic target of rapamycin (mTOR) pathways, respectively, has also been regarded as a possible up-stream regulator of the JNK pathway in gastric cancer cells [33,34]. Our results showed that SW-480 had the most prominent up-regulation of Akt phosphorylation under lower oxygen levels, while HT-29 was less activated. This could be due to the difference in phosphatase and tensin homolog (PTEN) status among the three cell lines. PTEN is a tumor suppressor and can inhibit Akt activation [35]. As PTEN has the highest expression in HT-29 [36], this causes the weakened Akt activation in HT-29 as shown in the result of this study. SP600125 surprisingly caused hypophosphorylation of Akt, especially for HT-29 and SW-480, in which Akt was previously considered as a possible upstream regulator of JNK in previous research [33]. This suggests a novel feedback mechanism of JNK-c-Jun-Akt for further promotion of EMT as a positive feedback mechanism and inhibition of autophagy through Akt-mTOR pathway as negative feedback mechanism by preventing simultaneous stimulation of autophagy through Akt-mTOR and JNK pathways.

There are numerous cancer stemness markers proposed in previous studies including pluripotency markers Oct-4, Nanog, and SOX2, which are considered as poor prognosis indicators of CRC [37,38]. Also, JNK may influence these markers through Wnt/BMP-TCF/Notch or IL-6-JAK-Stat3 pathways [15]. Oct-4 is the primary transcription factor required for stemness properties in CSCs. Overexpression of Oct-4, together or separately with other pluripotency markers such as Nanog, leads to tumor metastasis and recurrence in different cancer types [39]. From the results, both Oct-4 and Nanog were generally stimulated under lower oxygen levels, with HT-29 and DLD-1 having more prominent up-regulation. This verified both 1% and 10% O_2_ as possible stimulants of SM of CRC cells. In the presence of JNK inhibitor, the low oxygen level driven general up-regulations of Oct-4 and Nanog among the three cells lines were partially inhibited, except for Oct-4 in DLD-1. This suggests the importance of JNK in SM under low-oxygen environments, which could sequentially affect CSCs and the related tumorigenesis processes such as metastasis and radiosensitivity. The possible pathway could be Wnt/BMP-TCF/Notch or IL-6-JAK-Stat3 pathways as suggested in previous studies [15], which could be further confirmed in future studies.

Combining the results of this study, we showed the hypoxia-driven EMT induction/SM and proposed 10% O_2_ as another possible condition for EMT induction and SM among CRC cells. The blood oxygen level is about 10% in general. This study suggests that the regulation of EMT/SM and its related tumor progression events such as metastasis could be triggered by the oxygen level in blood. Moreover, the partial relieve of EMT induction and SM promotion by JNK inhibitor was demonstrated, and this suggests the role of JNK pathway mediation of EMT under low oxygen levels. Additionally, as the majority of CRC cells usually have null SMAD4 expressions in microsatellite instability (MSI)-negative CRC cell lines, including HT-29 and SW-480, but not for MSI-positive cell lines such as DLD-1, and this depends on SMAD-independent pathways such as JNK pathway [40,41], the JNK pathway has unique importance in EMT induction of CRC. Furthermore, this study proposes a novel pathway on the regulation of Akt by JNK-c-Jun, which could further control EMT and autophagy in CRC cells under low oxygen conditions. Inhibition of JNK is a potentially rewarding strategy for inhibiting EMT progression and SM in CRC cells. Further investigations with in vivo and clinical studies are recommended to establish JNK inhibition as a possible strategy to limit metastasis in CRC.

## 4. Materials and Methods

### 4.1. Cell Lines and Culture Conditions

Human colorectal adenocarcinoma cell line HT-29 was purchased from PerkinElmer, Inc. (Waltham, MA, USA). Human colorectal adenocarcinoma cell lines DLD-1 and SW-480 were obtained from Professor Jun Yu, Department of Medicine and Therapeutics, Faculty of Medicine, The Chinese University of Hong Kong (Hong Kong). All cells were cultured in DMEM medium with GlutaMAX supplement and HEPES and supplemented with 10% fetal bovine serum (FBS).

### 4.2. Hypoxic Condition and JNK Inhibition

The hypoxic and blood oxygen condition was maintained by the SCI-tive Dual chamber hypoxia workstation (Baker Ruskinn, Sanford, ME, USA) at 1% and 10% O_2_ with 5% CO_2_, 37 °C, and 100% humidity respectively. Cells cultured in normal incubator at an atmospheric oxygen level of 21% were used as the control.

For JNK inhibition, cells were treated for 48 h with 0.1% DMSO (control) or 10 µM of JNK inhibitor SP600125 (Sigma-Aldrich, St. Louis, MO, USA) dissolved in 0.1% DMSO as titrated in a preliminary experiment [32].

### 4.3. Cell Morphological Analysis

HT-29, DLD-1, or SW-480 cells (0.5 M) were seeded in T25 flasks. After overnight settling and 24 h serum starvation, cells were incubated in different O_2_ levels for 48 h with or without JNK inhibitor SP600125 (10 µM). The effect of oxygen level in EMT induction was evaluated by photomicrographs taken by light microscopy with 200× magnification. The cell morphology was visually compared and analyzed in 5 independent experiments.

### 4.4. Cell Migration Assay

Wound healing assay was employed to assess cell migration properties. Thirty-five thousand HT-29, DLD-1, or SW-480 cells were seeded in each compartment of a Culture-Insert 2 Well in µ-Dish 35 mm (Ibidi LLC, Munich, Germany). After overnight settling and 24 h serum starvation, the wound gap was made by removing the silicone insert. After that, fresh culture medium was added with or without JNK inhibitor SP600125 (10 µM), and cells were allowed to migrate under different oxygen levels for 24 h. Photomicrographs were taken before and after incubation. The gap area was measured using the MRI Wound Healing Tool macro for ImageJ software (NIH) (http://dev.mri.cnrs.fr/projects/imagejmacros/wiki/Wound_Healing_Tool). The invasion rate was calculated as the relative gap area difference between 0 and 24 h against the control among at least 3 independent experiments.

### 4.5. Immunofluorescence Staining

Fifty thousand HT-29, DLD-1, or SW-480 cells were seeded in 24-well plates with coverslips inserted. The serum starvation and incubation under different oxygen levels with or without JNK inhibitor SP600125 was the same as that of the cell morphological analysis. After incubation, cells were first gently washed by phosphate-buffered saline (PBS) for three times, then fixed with 4% paraformaldehyde (PFA) in PBS for 30 min. After washing by PBS for another three times, the cells were blocked with 2% bovine serum albumin (BSA) in PBS for 30 min. Alexa Fluor 594 Phalloidin (Thermo Fisher Scientific, Waltham, MA, USA) was added to the cells for staining F-actin in 1 h. Cells were mounted with ProLong Gold Antifade Mountant with 4′,6-diamidino-2-phenylindole (DAPI) (Thermo Fisher Scientific) after PBS washing. Cells and their cytoskeletons were visualized by confocal microscopy (Leica TCS SPE, Leica Microsystems, Wetzlar, Germany). The effect of oxygen level on cytoskeleton was quantified in terms of lamellipodia, filopodia, and ventral stress fibers. At least 200 cells were scored in each of the three independent experiments, and the percentages of cells having the three cytoskeleton structures of different conditions were plotted and compared.

### 4.6. Western Blotting

The cell density, incubation condition, and JNK inhibition were the same as that of the cell morphological analysis. Protein extraction and gel electrophoresis were performed as described in our previous paper [14]. The primary antibodies used are listed in Supplementary Table S1. The relative protein expression of each sample was evaluated by ImageJ software with β-Actin as loading controls. At least 4 independent sets were performed for each set of protein expression analyses. (All original western blot figures can be found in Supplementary Figures S2–S4)

### 4.7. Data Analysis

Different statistical tests have been used in analyzing the data. In general, values were plotted as mean ± SEM. Comparisons of means between independent groups were conducted by Student’s t test (2 groups) or Kruskal–Wallis one-way ANOVA (3 or more groups) with pairwise comparison. Comparison of mean between paired groups was conducted by paired t tests. Statistical analysis was conducted by Statistical Product and Service Solutions (SPSS) version 22 (IBM Corp, Armonk, NY, USA), and significance level of *p* < 0.05 was considered as statically significant.

## 5. Conclusions

It is clear that JNK, JNK pathway proteins, and transcription factors play important roles in EMT induction and SM of CRC cells under low-oxygen levels through multiple factors. Moreover, this study proposes a novel pathway of JNK-Akt serving as further control of EMT and other tumor progression processes such as autophagy. Thus, we suggest inhibition of JNK as a promising way for inhibiting EMT progression and SM in CRC cells.

## Supplementary Materials

The following are available online at https://www.mdpi.com/2072-6694/12/1/224/s1, Figure S1: Preliminary experiment of HT-29 wound healing assay; Figure S2: Original Western blots of Figure 4; Figure S3: Original Western blots of Figure 5; Figure S4: Original Western blots of Figure 6. Table S1: List of primary antibodies used in Western blotting.
