# Supplementary material for: JNK Pathway Mediates Low Oxygen Level Induced Epithelial–Mesenchymal Transition and Stemness Maintenance in Colorectal Cancer Cells
Source: Cancers (Basel). 2020 Jan 16;12(1):224. doi: 10.3390/cancers12010224 (PMC7017419; doi:10.3390/cancers12010224)
Supplement: Supplementary file 1 [file cancers-12-00224-s001.pdf]

cancers

MDPI

Article

JNK Pathway Mediates Low Oxygen Level-induced Epithelial-Mesenchymal Transition and Stemness Maintenance in Colorectal Cancer Cells

Shing Yau TAM, Vincent W.C. Wu and Helen K.W. Law

Table S1. List of primary antibodies used in Western blotting.

Primary Antibody (clones)	Source	Purchased from	Catalog Number
β-Actin (D6A8)	Rabbit	Cell Signaling Technology, Inc., Danvers, MA (CST)	8457
Akt	Rabbit	CST	9272
Phosphor-Akt (Ser473)	Rabbit	CST	9271
Phosphor-c-Jun (Ser73) (D47G9)	Rabbit	CST	3270
E-cadherin (24E10)	Rabbit	CST	3195
Fibronectin	Rabbit	Abcam, Eugene, OR	ab2413
Nanog (D73G4) XP	Rabbit	CST	4903
Nucleoporin 62 (SQSTM1/p62)	Rabbit	CST	5114
Oct-4	Rabbit	CST	2750
SAPK/JNK	Rabbit	CST	9252
Phosphor-SAPK/JNK (Thr183/Tyr185)	Rabbit	CST	9251
Snail (C15D3)	Rabbit	CST	3879
TWIST1	Rabbit	CST	46702
Vimentin (D21H3) XP	Rabbit	CST	5741

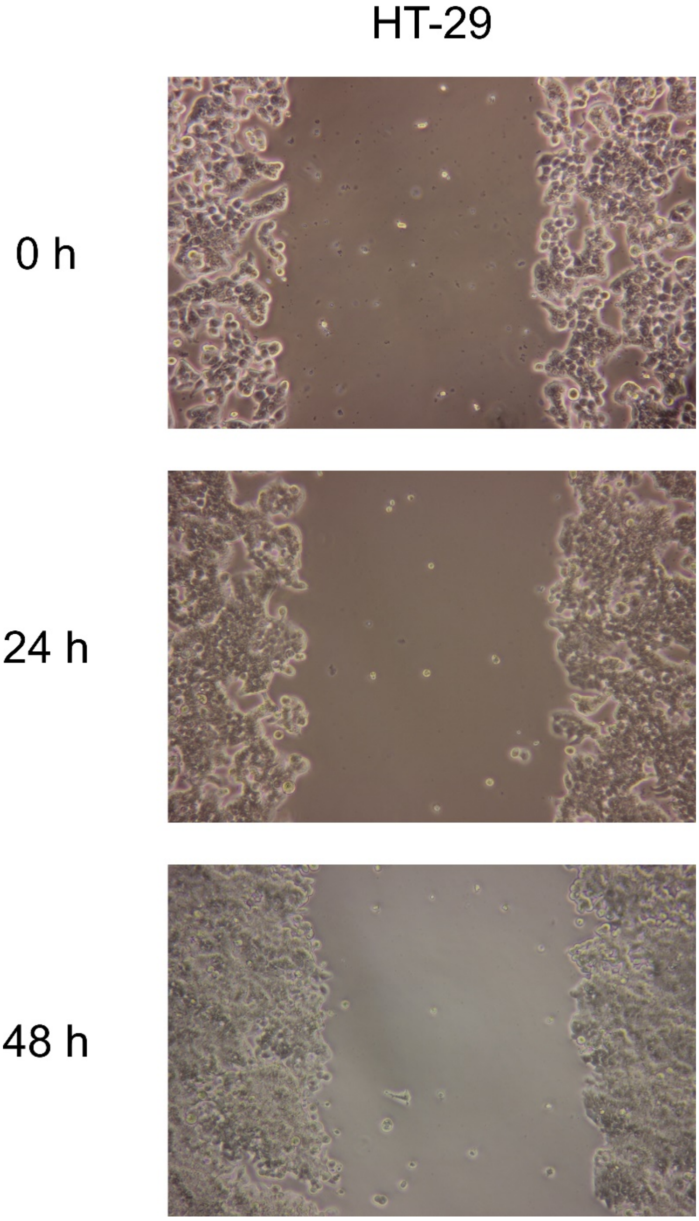

Figure S1. Preliminary experiment of HT-29 wound healing assay. The preliminary experiment shows that HT-29 has insignificant migration capacity in 24 or 48 hours under normoxia with 0.1% DMSO. Therefore, HT-29 was excluded in the wound healing assay in this study.

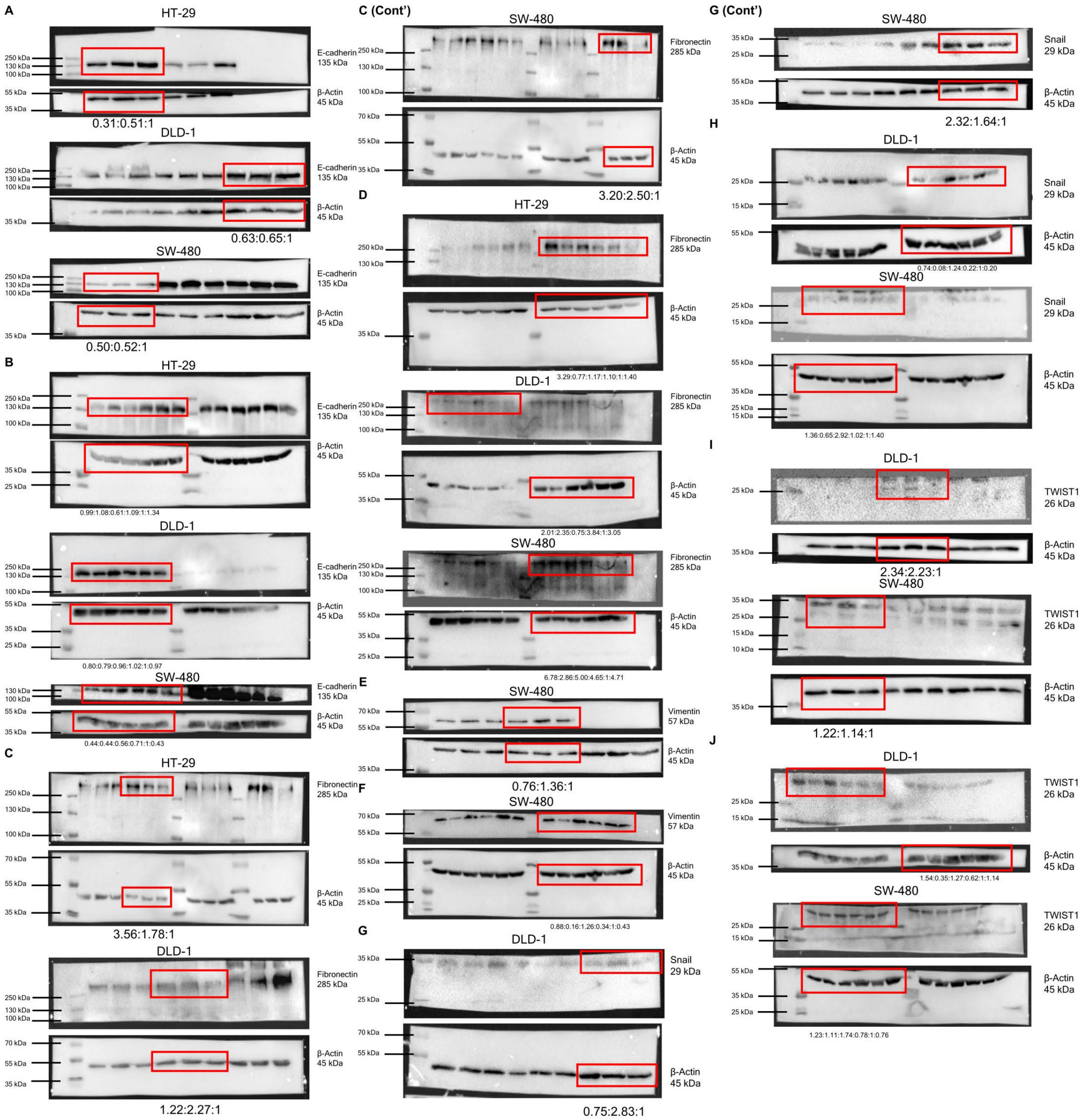

Figure S2. Original western blots of Figure 4.

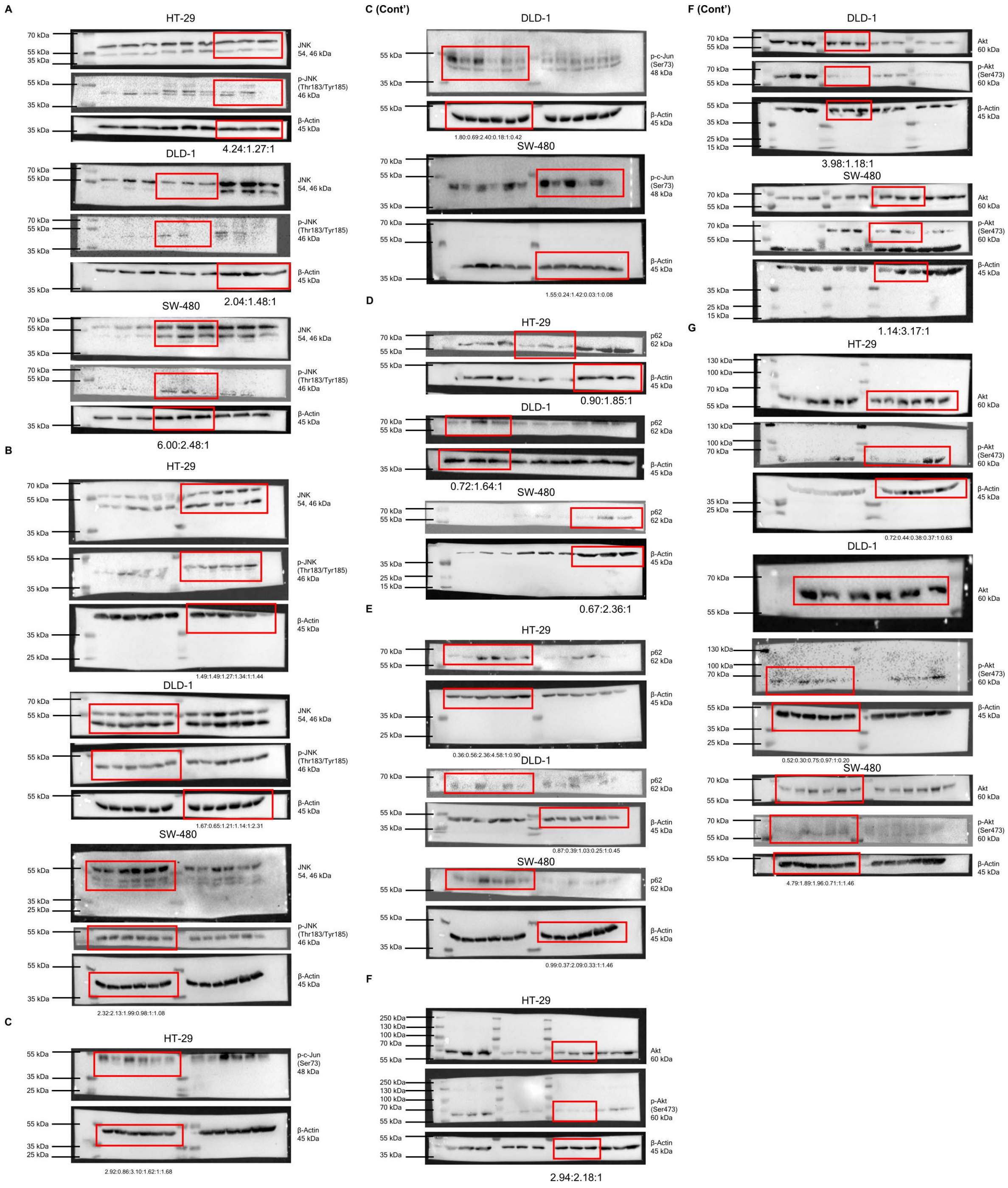

Figure S3. Original western blots of Figure 5.

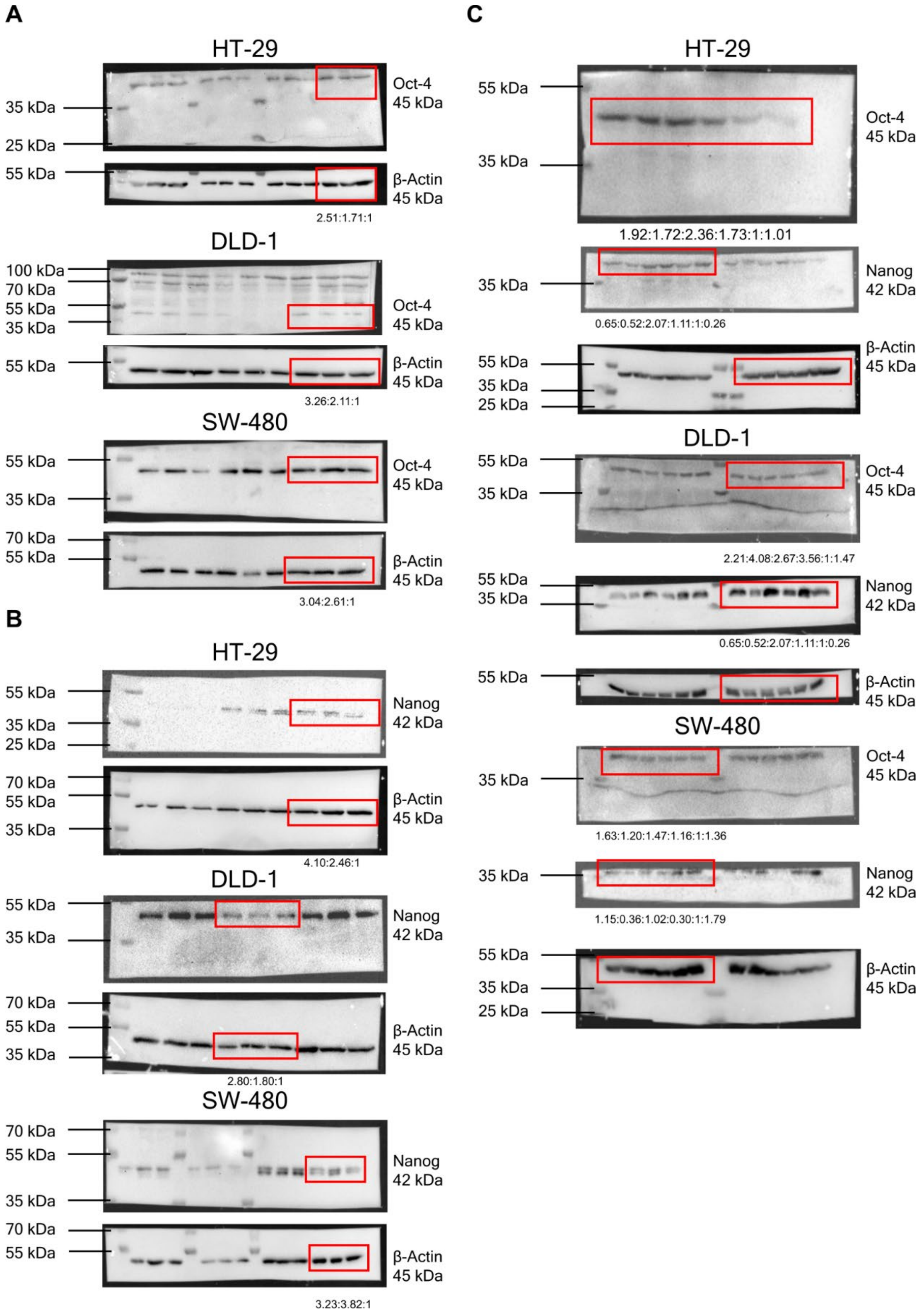

Figure S4. Original western blots of Figure 6.

© 2020 by the authors. Submitted for possible open access publication under the terms and conditions of the Creative Commons Attribution (CC BY) license (http://creativecommons.org/licenses/by/4.0/).
